# Association of bullying experiences with depressive symptoms and psychosocial functioning among school going children and adolescents

**DOI:** 10.1186/s13104-019-4236-x

**Published:** 2019-04-02

**Authors:** Sadiq Naveed, Ahmed Waqas, Kapil Kiran Aedma, Tayyaba Afzaal, Muhammad Hassan Majeed

**Affiliations:** 1 Kansas University Medical Center, Kanvas, USA; 2 CMH Lahore Medical College & Institute of Dentistry, Lahore Cantt, Pakistan; 3 Government College University, Lahore, Pakistan; 4 Natchaug Hospital, 189 Storrs Rd, Mansfield Center, CT 06250 USA

**Keywords:** Bullying, Depression, PHQ-9, Pakistan, Bully-victims, Distress

## Abstract

**Objectives:**

We examined the association of bullying experiences with depressive symptoms and psychosocial functioning among children and adolescents in rural Pakistan. A total of 452 school-going children in Nawabshah, Pakistan were conveniently interviewed to assess rates of bullying experiences and severity of depressive symptoms. Depressive symptoms were assessed using the Patient Health Questionnaire for Adolescents.

**Results:**

Experience of victimization was reported by 130 (28.8%) and perpetration by (146, 32.3%). A total of 162 (35.80%) reported mild depressive symptoms, 88 (19.50%) moderate, 33 (7.30%) moderately severe and 19 (4.20%) severe depressive symptoms. Age was not associated with patterns of bullying other than pure bully perpetration (.12, P = .024). Both victims and perpetrators of bullying experienced adverse emotional and social consequences. Bully-perpetrators exhibited the greater severity of depressive symptoms due to distress in psychosocial functioning.

**Electronic supplementary material:**

The online version of this article (10.1186/s13104-019-4236-x) contains supplementary material, which is available to authorized users.

## Introduction

Bullying is defined as a repetitive verbal, physical or psychological behavior by an individual intended to harm or disturb the victim with lesser power and control in the situation [1]. The behavior of bullying is prevalent in all culture, races, societies and settings such as school, work and even home USA [1, 2]. The prevalence of bullying among youth and adolescents also varies in different cultures for instance, it is estimated at 13% in Australia, 10% in Finland to as high as 30% in the USA [1–3]. This high prevalence is associated with several negative outcomes pertaining to the mental, social and physical health of the victim as well as the aggressor. These include depression, anxiety, loneliness, poor school attendance, academic under-achievement, social maladjustments and high-risk behaviors such as substance abuse, self-harm and suicide [1–4].

There are only a few studies in Pakistan exploring bullying behavior among school-going students, albeit reporting a significantly high prevalence (41%) [5]. This finding of high prevalence of bullying behavior in Pakistani culture is also corroborated by McFarlane et al. who reported alarmingly high rates of peer violence among Pakistani middle school students [6]. Their survey showed 94% of boys, 85% of girls have experienced victimization and 85% boys and 66% of girls have perpetuated violence towards peers [6]. School truancy, poverty, witnessing father’s aggression, gender role, the perception of masculinity and influence of patriarchal norms were among the psychosocial factors associated with bullying among the youth in Pakistan [6].

Despite being perceived as a major public health concern, only limited investigations have been conducted to explore bullying behavior among Pakistani school-going children. And this limited is exclusively in context of metropolitan cities in Pakistan. And there is a dearth of data about prevalence and outcomes of bullying behavior in rural Pakistan, where most of the population resides. Therefore, this study was designed to explore the prevalence of different patterns of bullying behavior and its association with depressive symptomatology and psychosocial among children and adolescents in rural Pakistan.

## Main text

### Methods

This cross-sectional study was conducted between September 2016 and July 2017 at a school situated in the city of Nawabshah. This study was approved by the ethical review board of the Peoples University of Medical and Health Sciences for Women, Nawabshah, Pakistan. A team of local researchers approached the administrative stakeholders of the school and sought their permission to conduct the study at the school site. Thereafter, an information packet containing the written informed consent form and a brochure regarding the study objectives was mailed to each child’s parents. Only those children were included who provided the written informed consent form. The parents were ensured anonymity and that only group level findings would be reported.

Children aged 10–17 years, studying at the school were part of the study population and were approached conveniently for interview by the researchers and their teachers. They were interviewed with a self-administered three-part survey containing items on (a) demographics, (b) experiences of bullying and victimization at school after 6 months (c) help-seeking behavior and (d) the Patient Health Questionnaire for Adolescents (PHQ-A). All survey items were in Urdu language; the national language of Pakistan.

Prior to the start of the study, the Patient Health Questionnaire for Adolescents was translated in Urdu language by experts in child and adolescent psychiatry [7]. It is a 9 items questionnaire pertaining to different symptoms of depression such as anhedonia that are rated on a four point Likert scale ranging from “not at all” to “almost every day” [8]. The scores on each individual item are then summed together to yield a global score, indicating the severity of depression, with a score of 20–27 considered as major depression [9]. The PHQ-A is also recommended for clinical evaluation of depression and research in Section III of the DSM-V [10].

The present study used the impact supplement of the Strengths and Difficulties Questionnaire-Urdu translation (SDQ) to assess frequency of distress among participants at their homes, friendships, and leisure and classroom activities [11]. The responses on the impact supplement were summed together to yield a global scores representing overall distress in life.

Participants reported the frequency of both victimization and perpetration behaviors. These items provided details on bullying behavior at school and outside school, e.g. at private tuition centers, in last 6 months. The responses to these four items were recorded on a Likert-type scale ranging from “not at all” to “most days”. These items yielded good internal consistency (α = .820).

All data were analyzed using SPSS (v.20). Descriptive statistics were presented for quantitative and categorical variables. A series of multiple linear regression analyzes was used to delineate bullying behavior as predictors of depression and distress in psychosocial functioning. Prior to running regression analyzes, assumptions regarding multicolinearity were assessed using variance inflation factor and tolerance statistics. Mediating effects of distress in psychosocial functioning on the relationship between depressive symptoms and bullying behavior was analyzed using PROCESS software [12].

### Results

There were a total of 452 respondents (response rate = 90.40%), majority girls (364, 80.50%) with a mean age of 13.93 (1.78) years. All of the respondents were enrolled in Nawabshah city. A total of 162 (35.80%) reported mild depressive symptoms, 88 (19.50%) moderate, 33 (7.30%) moderately severe and 19 (4.20%) severe depressive symptoms. Mean scores on PHQ-A scale were 7.5 (SD 5.57).

Among these respondents, there were a total of 70 (15.50%) bully victims, 60 (13.30%) pure victims, and 76 (16.80%) pure bully perpetrators. A total of 20.6% of the respondents reported being bullied by girls and 10.7% by boys at least once a week. A total of 21.5% of the respondents were bullied more than once a week in past 6 months and away from school (13.1%). While 27.7% of the children and adolescents bullied others at least once a week in past 6 months at school and away from school (14%). Chi-square test of association did not reveal any significant association between gender and pattern of bullying experiences (P > .05). Age was not associated with patterns of bullying: bully-victims (r = − .08, P = .09), pure victims (r = − .03, P = .48), while it was significantly associated with pure bully perpetrators (0.12, P = .024).

A total of 138 (30.5%) did not feel the need to get help from outside for their negative feelings, and emotional problems, while 84 (18.6%) had considered getting help and 230 (50.9%) reported seeking help. A total of 145 (32.1%) preferred friends, 122 (27%) parents, 46 (10.2%) teachers, 28 (6.2%) siblings, and 7 (1.5%) preferred relatives for help, while 104 (23%) did not report any preference.

A total of 72.7% of the respondents experienced emotional or behavioural difficulties with the majority (66.6%) experiencing it for less than month. A majority (67%) reported being distressed by these difficulties with a majority experiencing these in several facets of life including home life, friendships, classroom learning and leisure activities (Additional file 1: Table S1).

Multiple linear regression explained 10.30% of variance in depression symptoms (F_(451)_ = 11.36, P < .001). Depression scores were positively associated with age, victimization, perpetration as well as bully victimization. Experience of bully victimization was the strongest predictor of depressive symptomatology followed by perpetration and victimization (Table 1). Distress in life (F_(451)_ = 3.76, P = .002) was associated with bully-victims and victimization but not perpetration (Table 1).

**Table 1:** Predictors of depression scores and psychosocial functioning among school going children (n = 452)

Variables	Unstandardized coefficients		Standardized coefficients Beta	*t*	*p*
	B	Std. error			
Depression severity					
(Constant)	− 4.426	3.336		− 1.327	.185
Gender	1.208	.633	.086	1.908	.057
Age	.605	.214	.128	2.826	.005
Bull victim	4.901	.717	.319	6.837	.000
Pure victim	1.951	.760	.119	2.567	.011
Pure bully	1.803	.696	.121	2.591	.010
Psychosocial functioning					
(Constant)	6.023	1.929		3.123	.002
Gender	.602	.366	.077	1.645	.101
Age	.084	.124	.032	.682	.496
Bully victims	1.547	.414	.181	3.733	.000
Pure victims	.912	.439	.100	2.076	.038
Pure perpetrators	.758	.402	.092	1.883	.060

Distress in psychosocial functioning exerted significant mediation effects on relationship between depression scores and bully-victimization with an indirect effect size of 1.05 (95% CI .38–1.78). While no significant mediation effects of distressed functioning were noted for pure perpetrators and victims.

### Discussion

Bullying-related behavior (victimization, bully-victims and perpetration), at or away from school, were highly prevalent among children and adolescents in our study. Both victims and perpetrators of bullying experienced adverse emotional and social consequences of the behavior. Distress in psychosocial functioning accounted for the greatest severity of depressive symptoms among bully-perpetrators than victims and bullies alone.

The prevalence of bullying behavior in our study sample was higher than those in the United States study where 13% identified as a bully, 10.6% as a victim and only 6.3% as both [1]. In a recent survey in Australia, 13.3% were bully-victims, 1.6%, perpetrators and 1.9% both [1–4] while in Finland, the overall bullying behavior was only around 10% [2]. In contrast to other studies, in this sample females were more likely to be the perpetrators than males. However, the present analyzes reveal a much lower prevalence of bullying behavior when comparing with previous studies in Pakistan [5, 6]. These differences in experience of victimization and perpetration of bullying may be due to study sample restricted to one academic year [6] or a narrow age group [5], and differences in measurements of bullying behavior. Moreover, previous studies did not account for bully-victims, exhibiting both victimization and perpetration behavior and rather, pooled them into a dichotomous classification of victims and bullies.

In present study, bully-victimization and victimization but not perpetration were associated with distress in life including poor functioning in the area of schools, relationship with friends, family bonding and participation in leisurely activities. Our findings partially corroborate those of an earlier global study by Nansel et al. who reported bullying victims exhibited worsening psycho-social adjustment when compared with bully aggressors [4].

Our survey showed depression was most significantly associated with bully-victimization but also to perpetuation and victimization. Bully-victims showed a particularly high risk and vulnerability for depressive symptoms than their counterparts. The association of depression with bullying behavior is already well documented in the medical literature [2]. Our finding confirms this for the Pakistani population as well. The greater association of depressive symptoms with bully-victims was due to mediational effects of psychosocial disturbances which were particularly high in this group.

### Conclusion

These findings have public health value, and present a significant association between depressive symptoms among children who have experienced bullying. Bully-victims report a higher preponderance toward depression than pure victims and perpetrators and this relationship is mediated by distress in psychosocial functioning. These findings may help in designing policies and interventions, taking into consideration that comorbid victimization and perpetration behaviors are quite common and account for higher distress in daily lives.

## Limitations

There are several strengths of this study. It is the first report of bullying behavior among school-going children in a peripheral and poorly developed region in Pakistan. Adequate sample size and use of valid and reliable scales for assessing depression and psychosocial functioning are particular strengths. Despite these strengths, this study is limited by its cross-sectional design is establishing causality. Moreover, this study was conducted at one school in a city of Pakistan, therefore, these findings should be generalized with caution. Mediation analyses are more appropriate with longitudinal study designs, therefore, the finding of a mediation relationship between bullying, distress and depression is exploratory in nature, and should be interpreted with caution [13].

## Additional file

**Additional file 1: Table S1.** Pattern of bullying experiences among the respondents (n = 452). It provides detailed statistics on pattern of bullying experiences and distress among children participating in the study.

## Abbreviations

PHQ-A: Patient Health Questionnaire for Adolescents
SDQ: Strengths and Difficulties Questionnaire-Urdu translation
