# Supplementary material for: Association of bullying experiences with depressive symptoms and psychosocial functioning among school going children and adolescents
Source: BMC Res Notes. 2019 Apr 2;12:198. doi: 10.1186/s13104-019-4236-x (PMC6444870; doi:10.1186/s13104-019-4236-x)
Supplement: Supplementary file 1 — Additional file 1: Table S1. Pattern of bullying experiences among the respondents (n = 452). It provides detailed statistics on pattern of bullying experiences and distress among children participating in the study. [file 13104_2019_4236_MOESM1_ESM.docx]

Additional table S1: Pattern of bullying experiences among the respondents (n=452)

| Statements | Response | Frequency (n) | Percentage (%) |
| --- | --- | --- | --- |
| How often have you been bullied in school in the past six months? | Not at all | 355 | 78.5% |
|  | Less than once a week | 45 | 10.0% |
|  | More than once a week | 28 | 6.2% |
|  | Most days | 24 | 5.3% |
| How often have you been bullied away from school in the past six months? | Not at all | 393 | 86.9% |
|  | Less than once a week | 39 | 8.6% |
|  | More than once a week | 10 | 2.2% |
|  | Most days | 10 | 2.2% |
| How often have you been bullying others in school in the past six months? | Not at all | 327 | 72.3% |
|  | Less than once a week | 82 | 18.1% |
|  | More than once a week | 20 | 4.4% |
|  | Most days | 23 | 5.1% |
| How often have you been bullying others away from school in the past six months? | Not at all | 387 | 86.0% |
|  | Less than once a week | 35 | 7.8% |
|  | More than once a week | 17 | 3.8% |
|  | Most days | 11 | 2.4% |
| Bullied by girls | Not at all | 359 | 79.4% |
|  | Less than once a week | 29 | 6.4% |
|  | More than once a week | 16 | 3.5% |
|  | Most days | 48 | 10.6% |
| Bullied by boys | Not at all | 402 | 89.3% |
|  | Less than once a week | 17 | 3.8% |
|  | More than once a week | 13 | 2.9% |
|  | Most days | 18 | 4.0% |
| Bullied by a group | Not at all | 419 | 92.9% |
|  | Less than once a week | 8 | 1.8% |
|  | More than once a week | 9 | 2.0% |
|  | Most days | 15 | 3.3% |
| Have you told someone about your experience so that they can help you? | Yes | 270 | 59.7% |
|  | No | 182 | 40.3% |
| Overall, do you think that you have difficulties in one or more of the following areas: emotions, concentration, behaviour or being able to get on with other people? | No | 121 | 27.3% |
|  | Yes, minor difficulties | 238 | 53.8% |
|  | Yes, definite difficulties | 58 | 13.1% |
|  | Yes, severe difficulties | 25 | 5.7% |
| How long have these difficulties been present? | Less than a month | 299 | 66.6% |
|  | 1-5 months | 73 | 16.3% |
|  | 6-12 months | 22 | 4.9% |
|  | Over a year | 55 | 12.2% |
| Do the difficulties upset or distress you? | Not at all | 149 | 33.0% |
|  | Only a little | 195 | 43.2% |
|  | Quite a lot | 68 | 15.1% |
|  | A great deal | 39 | 8.6% |
| Home life | Not at all | 234 | 51.8% |
|  | Only a little | 149 | 33.0% |
|  | Quite a lot | 50 | 11.1% |
|  | A great deal | 19 | 4.2% |
| Friendships | Not at all | 246 | 54.5% |
|  | Only a little | 143 | 31.7% |
|  | Quite a lot | 38 | 8.4% |
|  | A great deal | 24 | 5.3% |
| Classroom learning | Not at all | 270 | 59.7% |
|  | Only a little | 118 | 26.1% |
|  | Quite a lot | 40 | 8.8% |
|  | A great deal | 24 | 5.3% |
| Leisure activities | Not at all | 219 | 48.5% |
|  | Only a little | 121 | 26.8% |
|  | Quite a lot | 64 | 14.2% |
|  | A great deal | 48 | 10.6% |
| Do the difficulties make it harder for those around you (family, friends, teachers, etc.)? | Not at all | 342 | 75.7% |
|  | Only a little | 82 | 18.1% |
|  | Quite a lot | 20 | 4.4% |
|  | A great deal | 8 | 1.8% |
